# Early migration following revision total knee arthroplasty with tibial metaphyseal cones: a 2-year prospective RSA cohort study of 25 patients

**DOI:** 10.2340/17453674.2026.45964

**Published:** 2026-06-10

**Authors:** Kelly MILLS, Maartje BELT, Jon H M GOOSEN, Gijs G VAN HELLEMONDT, Petra J C HEESTERBEEK

**Affiliations:** Sint Maartenskliniek, Nijmegen, the Netherlands

## Abstract

**Background and purpose:**

In revision total knee arthroplasty (rTKA), cones can enhance stable fixation in the case of suboptimal metaphyseal bone stock. Combined with stems of sufficient length, cones can offer support and promote osseointegration without graft-related complications. However, their mechanical stability remains underexplored. We aimed to evaluate the stability of tibial rTKA constructs with metaphyseal cones using radiostereometric analysis (RSA) over a 2-year follow-up.

**Methods:**

25 patients undergoing rTKA with a tibial cone were included. Tantalum markers were placed in the cancellous bone intraoperatively, before placement of the revision construct, to enable migration measurements using model-based RSA (MB-RSA). Tibial component migration was assessed at baseline, 6-week, 3-month, 6-month, 1-year, and 2-year follow-up, with main outcome parameters being total translation (TT) and total rotation (TR) at 2-year follow-up. Clinical outcomes included VAS pain and satisfaction, the Oxford Knee Score, Knee Society Score, KOOS-PS, and adverse events.

**Results:**

At 2-year follow-up the least-squares mean TT was 0.59 mm (95% confidence interval [CI] 0.40–0.78) and TR was 0.76° (CI 0.57–0.96). Migration measures demonstrated substantial inter-individual variability, with heterogeneous early migration patterns across patients. Between 1 and 2 years, median changes in migration were small at group level. At 2 years, 3 of 23 patients had TT > 1 mm and 6 had TR > 1°. Clinical outcome scores generally improved until 6 months to 1 year, followed by a trend towards decline. There was 1 re-revision for aseptic loosening after 1-year follow-up and 1 insert exchange for knee instability.

**Conclusion:**

Tibial rTKA constructs with metaphyseal cones demonstrate heterogeneous early migration, followed by limited additional migration at group level between 1 and 2 years. While most constructs showed low migration at 2 years, several patients exhibited increased migration.

Achieving durable fixation of revision total knee arthroplasties (rTKAs) is challenging due to compromised bone stock and bone loss resulting from the removal of the previous implant. According to Morgan-Jones et al., stability in at least 2 of the 3 bone zones (i.e., the epiphysis, metaphysis and diaphysis) is essential to achieve durable fixation [1]. More recently, van Laarhoven et al. emphasized the critical role of the metaphyseal zones in revision TKA [2].

Various techniques exist to improve metaphyseal fixation in cases of bone loss. Biological reconstruction techniques like bone impaction grafting or bulk structural allografts aim to restore bone stock but may have limited initial stability, risk of graft failure, and technical complexity. In contrast, the use of metaphyseal cones or sleeves offers immediate mechanical metaphyseal support and surgical simplicity, but may lead to stress-shielding, which can reduce bone density and potentially compromise bone health [3]. However, they might also enable the use of shorter stems, reducing diaphyseal anchorage and related complications, while enhancing load distribution and fixation surface. This makes them a reliable option in complex revisions.

Although previous studies demonstrated signs of osseointegration of rTKA with cones [4,5] and reported relatively low revision rates in the short and long term [6-8], none assessed implant stability using more precise methods. Radiostereometric analysis (RSA) provides a highly accurate method for assessing implant migration, with early excessive migration shown to predict future aseptic loosening [9]. As such, RSA enables early identification of at-risk implants, providing predictive value for long-term fixation without extended follow-up [10].

Our primary aim was to assess tibial component migration in rTKA using metaphyseal cones, quantified by RSA up to 2 years. The secondary aim was to evaluate clinical outcomes.  

## Methods

**Figure 1:**
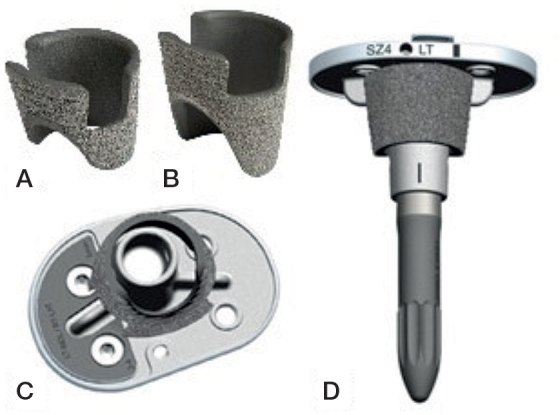
Components of the metaphyseal cone–augmented revision TKA construct. A–B:.(A) Short and (B) long Legion titanium tibial cone with STIKTITE coating. C. Tibial baseplate with cone interface (undersurface view). D. Total tibial Legion Revision construct with cone and stem assembled. Image courtesy of Smith & Nephew, USA.

This cohort study was performed from November 2019 until March 2025 at the Sint Maartenskliniek, Nijmegen, the Netherlands. This study was conducted and reported in accordance with the Declaration of Helsinki [11], ISO 16087:2013 for RSA, RSA [12], and STROBE guidelines [13]. All participants were assigned to undergo either first revision (after TKA) or re-revision TKA, with the Legion Revision Total Knee Arthroplasty System (Legion rTKA System, Smith & Nephew, Memphis, TN, USA) combined with metaphyseal cones (Figure 1; Smith & Nephew, USA) because of metaphyseal bone loss. The indication for cone use was established preoperatively based on metaphyseal bone loss assessed on routine radiographs. The definitive decision to implant a cone was confirmed intraoperatively following direct evaluation of metaphyseal bone quality by the treating orthopedic surgeon. Exclusion criteria were: age > 78 years at the time of the surgery, ASA score 4, untreated cardiac, pulmonary, hematological, or other conditions that pose excessive operative risk, indication for a hinged-type revision system (in case of insufficiency of the collateral ligaments or the extensor mechanism), physical, emotional, or neurological conditions that would compromise the patient’s compliance with postoperative rehabilitation protocol follow-up, active local or systemic infection, immunosuppressive disorders (e.g., rheumatoid arthritis), and/or known sensitivity to materials used in the device. All patients were recruited by a research nurse and the treating orthopedic surgeon.

### Surgical procedure

All patients received the Legion revision TKA system; instrumentation was used according to the surgical technique brochure by Smith & Nephew [14]. All patients received spinal anesthesia or regional femoral and sciatic nerve blocks. Surgery was performed using the medial parapatellar approach. All procedures were performed by 6 experienced orthopedic surgeons. In case of a press-fit stem, a 120 mm stem with an offset coupler was applied. When an offset coupler was not warranted, a 160 mm stem was used. In the situation that a press-fit stem could not be used (i.e., small cone, tibial alignment, and/or diaphyseal bone loss), cemented stems were used. In cases with a cemented stem, a 120 mm stem without offset coupler was used after 2 mm over-reaming. The tibial plateau was cemented with dual-antibiotic bone cement (COPAL G+C, Heraeus Medical, Germany). For hybrid fixation, cement was applied to the tibial plateau, offset coupler, and the inner surface of the cone, while the press-fit stem was left uncemented. All cones were implanted press-fit with full bone contact at the outer surface, leaving no cement interposition between cone and host bone to ensure optimal fixation. The cones (Legion, Smith & Nephew, USA; Figure 1) had a pore size of 200 μm and a porosity of 60%. During the surgery tantalum 1.0 mm RSA beads (Halifax Biomedical Inc, Halifax, NS, Canada) were inserted to form the bone model for RSA analysis. Those markers were placed after preparation of the tibial bone and rinsing of the surface and were placed in the cancellous bone at the end of the metaphysis (near the diaphysis). All native patellae received a button (n = 16); if already resurfaced, the button was carefully checked for loosening and/or wear and revised when necessary. Tuberosity osteotomy was performed when necessary (n = 4). Additional bone grafts (n = 3) and/or tibial wedges (n = 9; Legion, Smith & Nephew) were used by indication. No release of the lateral retinaculum was performed. All procedures were performed in 1 stage. The amount of bone loss was scored intraoperatively as none, mild, moderate, or severe, for the epiphysis, metaphysis, and diaphysis separately, according to the classification by Belt et al. [15].

### Implant migration

Fixation of the implant was measured as implant migration using RSA. Radiographs were made with patients in a supine position, in a uniplanar setup with 1 ceiling-mounted and 1 mobile radiograph tube. The radiographic images were made at baseline (max. 3 days after surgery, after mobilization of the patient), 6 weeks, 3 months (± 2 weeks), 6 months (± 2 weeks), 1 year (± 1 month), and 2 years (± 2 months) postoperatively. Digital radiograph detectors were used (Skyplate Large, Philips, Amsterdam, the Netherlands) with an image resolution of 172 dpi. All RSA measurements were performed using the MBRSA software (MBRSA version 4.2014, RSAcore, Leiden, the Netherlands). The same marker-model was used across all time points. Generally, a condition number (CN) below 120 was used as described in the guidelines, otherwise a marker configuration model (MCM) was made (n = 8) [16]. In some cases (with suboptimal distribution of markers, or occluded markers on all RSA images), when an MCM could not improve the CN, a higher CN was accepted, up to a maximum of 180. In these cases, all markers in the model were thoroughly checked for reliability and fixation and analyses were checked by multiple analysts. Cases with CN higher than 180 were excluded from the analysis. In accordance with the RSA guidelines [12], an upper limit of 0.35 mm for the mean error of rigid-body fitting (ME) was applied.

**Figure 2:**
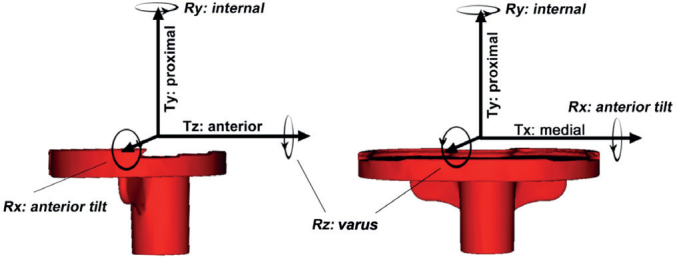
Orientation of the 3 axes (longitudinal [Y], transverse [X], and sagittal [Z]) and directions of translations and rotations on and around these axes. T: Translation; R: Rotation. Directions apply to a right tibial component in a right-hand coordinate system relative to the tibial bone. Edited version of the original image from Mills et al. [24].

Tibial component migration was determined relative to the tibial bone model and expressed translation (T) along and rotation around (R) the 3 axes (Figure 2). It was assumed that the tibial component formed a fixed construct with the cone and stem. The measurement error (precision) was determined using all available double examinations (n = 20) at 6 weeks postoperatively (with 1 exception where the double examinations were done at 3-month follow-up), by using the first radiograph as a reference and calculating the implant migration of the 2nd radiograph compared with the reference. For each double examination, the patient was repositioned and the RSA setup fully rebuilt prior to the second radiograph to ensure an independent repeated measurement. The precision of the migration measures is presented in Table 1. For the longitudinal RSA measurements, the 1st image of the double examinations was used, unless it was invalid, in which case the second image was used (n = 1).

**Table 1:** Precision of RSA measurements for tibial constructs, assessed through double examinations. Precision was calculated by using the first image as the reference and determining the migration of the second image relative to the first.

Axes	Migration	
	mean (SD)	median (IQR
Translation (mm)		
X	–0.01 (0.10)	–0.01 (–0.0 to 0.07)
Y	0.00 (0.04)	0.00 (–0.03 to 0.03)
Z	0.01 (0.19)	–0.02 (–0.12 to 0.09)
Total	0.18 (0.11)	0.17 (0.10 to 0.23)
Rotation (°)		
X	0.01 (0.17)	0.01 (–0.13 to 0.09)
Y	0.00 (0.43)	–0.04 (–0.27 to 0.11)
Z	–0.01 (0.09)	–0.02 (–0.04 to 0.04)
Total	0.39 (0.25)	0.33 (0.24 to 0.44)
MTPM	0.39 (0.20)	0.38 (0.23 to 0.52)

Total Translation = Euclidean sum of X, Y, and Z translation.

Total Rotation = Euclidean sum of X, Y, and Z rotation.

MTPM: Maximal Total Point Motion.

### Clinical and functional outcomes

To evaluate clinical and functional outcomes, both patient-reported (PROMs) and clinician reported outcome measures (CROMs) were used. Pain and satisfaction were assessed using Visual Analog Scale (VAS) scores, ranging from 0 to 100, with 0 indicating no pain or the lowest satisfaction. The Oxford Knee Score was used, with scores ranging from 0 to 48, where 48 represents the best outcome [17]. The Knee Society Score (KSS) [18] was obtained, comprising both a clinical and functional score, each ranging from 0 to 100. Additionally, the Knee Injury and Osteoarthritis Outcomes Score – Physical function Short form (KOOS-PS) [19] was collected, ranging from 0 to 100. Dutch-validated versions were used. All device-related adverse events (AEs) were noted.

To aid clinical interpretation, changes in PROMs were compared with established minimal clinically important difference (MCID) thresholds. MCIDs specific to revision TKA were applied where available (OKS = 4.9 points [20], KSS functional = 6.3 points, KSS clinical = 6.6 points [21]), and MCIDs derived from primary TKA cohorts were used for VAS pain (–2.3 points [22]) and KOOS-PS (2.2 points MCID and 15.0 points for moderate improvement [23]) due to limited revision-specific data. These thresholds were used descriptively and not for hypothesis testing.

### Statistics

No formal sample size calculation was performed as no comparative group or benchmark was available. A target of 25 participants was chosen based on feasibility and ethical approval. Previous RSA studies have shown that reliable results can be obtained with relatively small cohorts (15–25 patients) due to the high measurement accuracy of the method [12].

Patient characteristics were summarized using mean (standard deviation [SD], range) for continuous data (these were normally distributed) and counts for categorical variables. RSA outcomes and patient- and clinician-reported outcome measures were summarized descriptively at each follow-up time point using both median (interquartile range [IQR]) and Least-Squares (LS) means (95% confidence interval [CI]) derived from Mixed Model for Repeated Measures (MMRM) analyses. This approach was chosen to provide a complete overview of the data distribution while remaining consistent with reporting conventions. MMRM uses all available follow-up time points, accounts for within-patient correlation across repeated measures, and appropriately handles missing data under the missing-at-random assumption.

All RSA outcomes were reported as Total Translation (TT = √(Tx^2^ + Ty^2^ + Tz^2^)) and Total Rotation (TR = √(Rx^2^ + Ry^2^ + Rz^2^)). For comparison with previous literature the Maximum Total Point Motion (MTPM), the points on the femoral and tibial component that move the most, was also reported. Because no validated threshold for clinically relevant migration exists, a limit of > 1 mm TT or > 1° TR was applied. This benchmark was chosen arbitrarily, in line with previous RSA research [24], to reflect migration exceeding measurement precision and to identify implants that may warrant closer clinical follow-up. Migration was considered stable when the ΔMTPM_6–12 months_ (early stabilization) or ΔMTPM_1–2 years_ was below 0.2 mm.

To evaluate implant migration over time, MMRM was applied with Tx, Ty, Tz, TT, Rx, Ry, Rz, TR, and MTPM as dependent variables and time (categorical) as fixed effect. Patient ID was specified as the repeated subject, and an unstructured covariance matrix was used to account for within-subject correlation. To explore potential associations between construct-related factors (cone length, stem length, tibial wedges, bone grafting, insert type) and migration outcomes at 2-year follow-up, exploratory univariate regression analyses were performed. Variables showing a significant association with TT or TR at 2 years were subsequently entered as covariates in an extended MMRM. The main outcome parameters were the TT and TR at 2-year follow-up, estimated from the MMRM using least squares means (LS means) and 95% CI. MTPM means were also reported similarly, for comparison with previous studies.

All analyses were performed using R (version 4.5.1; R Foundation for Statistical Computing, Vienna, Austria) in RStudio (version 2025.09.1; Posit Software, Boston, MA, USA). A P-value < 0.05 was considered statistically significant where applicable.

### Ethics, funding, data sharing, and disclosures

Ethical approval for this study was obtained from CMO Regio Arnhem-Nijmegen (NL66833.091.18), the study was registered in the Dutch Trial Register (https://www.onderzoekmet-mensen.nl/nl/trial/52619) and all patients provided written informed consent. The study was funded by Smith & Nephew, but the company had no role in the design or conduct of the study, the collection, management, analyses, and interpretation of the data. The datasets generated and analyzed during the current study are not publicly available due to privacy regulations under the Dutch General Data Protection Regulation (AVG), but anonymized output data is available from the corresponding author upon reasonable request. Please note that original radiographic images and patient-identifiable information cannot be shared due to privacy regulations and ethical considerations. However, RSA-derived data and analysis outputs underlying the findings are available for academic purposes.

JG has received fees or honoraria from Smith & Nephew and Stryker and GGvH from ZimmerBiomet, Smith & Nephew, and Materialise. Complete disclosure of interest forms according to ICMJE are available on the article page, doi: 10.2340/17453674.2026.45964

## Results

**Figure 3:**
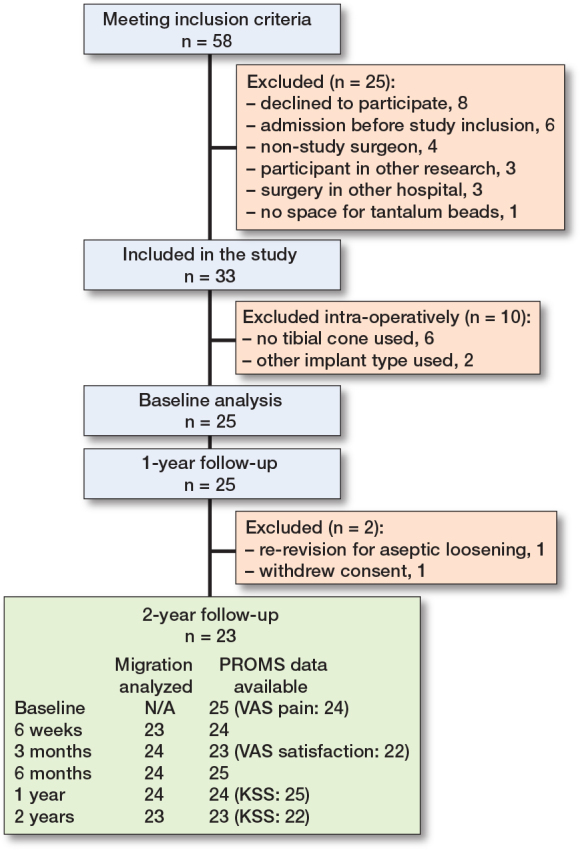
Patient flow diagram illustrating enrollment, exclusions, and follow-up completion.

Figure 3 presents the flowchart for exclusion and follow-up, with the pre- and intraoperative exclusion counts and corresponding reasons. Eventually 25 patients were included, of whom 23 completed 2-year follow-up as 1 patient withdrew consent from further follow-up and 1 re-revision was performed, both after 1-year follow-up. All patients and their implant construct characteristics can be found in Table 2.

**Table 2:** Baseline patient and implant construct characteristics. Values are mean (standard deviations) and range or count

Factor	Baseline values (n = 25)
Age (years)	65.3 (6.1) 55–77
Sex (female/male)	17/8
Body mass index	29.8 (4.7) 21.6–37.0
Revision stage (first revision/re-revision)	15/10
Previous fixation type	
first revision (cemented/uncemented)	14/1
re-revision (hybrid/fully cemented)	9/1
Side (left/right)	14/11
Bone loss (none/mild/moderate/severe) **^a^**	
epiphysis	0/1/10/14
metaphysis	1/5/17/2
diaphysis	15/9/1/0
Fully cemented/hybrid fixed stems	23/2
Tuberosity osteotomy (yes/no)	4/21
Tibial wedges (yes/no)	9/16
Insert type (PS/constrained) **^b^**	7/17
Cone (short 25 mm/long 40 mm)	18/7
Stem length (120 mm/160 mm)	17/8

PS: Posterior-stabilized.

a Bone loss classification according to Belt et al. [15].

b 1 unknown insert type.

### Implant migration

**Figure 4:**
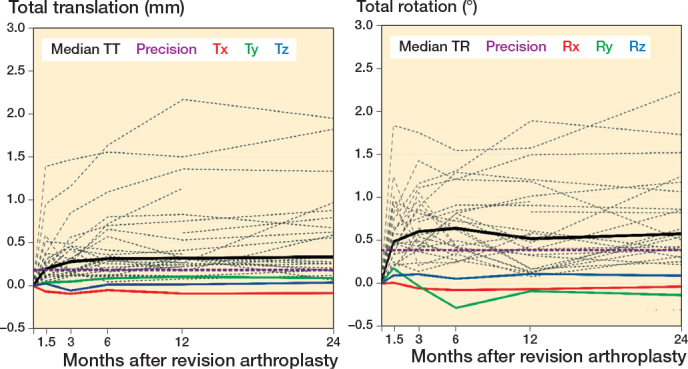
Median translations (in mm) (left panel) and median rotations (in degrees) (right panel) of the tibial component along and around the x, y, and z axis over follow-up time, as well as the corresponding median total translation (TT), median total rotation (TR), median TT precision, and median TR precision. The dashed lines present the individual participants’ total translations.

The LS mean TT and TR at 2-year follow-up were 0.59 mm (CI 0.40–0.78) and 0.76° (CI 0.57–0.96), respectively. A complete overview of all migration measures is provided in Table 3. The median ΔMTPM_6–12 months_ and ΔMTPM_1–2 years_ were –0.02 mm (IQR –0.51 to 0.98) and 0.10 mm (IQR –0.54 to 1.11), respectively. Figure 4 present the median translational and rotational migration patterns over time, illustrating the typical migration behavior of the group, as well as individual patient trajectories. At 2-year follow-up there were 6 patients with a TR > 1° (out of 23) and 3 of them also had TT > 1 mm. 1 patient with a TT > 1 mm (TR just below 1°) at 1-year follow-up was re-revised. Detailed data for patients exceeding these thresholds is provided in Supplementary Table S1. At the patient-level, 8 patients demonstrated a ΔMTPM_1–2 years_ exceeding 0.2 mm.

**Table 3:** Median (IQR) and least-square (LS) means with 95% confidence interval (CI) migration (translation in mm and rotation in °) of the tibial component with regard to the tibial bone model at all follow-up time points referenced to the baseline measurement directly (max. 3 days) after surgery

Item	6 weeks (n = 23)	3 months (n = 24)	6 months (n = 24)	1 year (n = 24)	2 years (n = 23)
X translation, mm					
Median (IQR)	–0.07 (–0.17 to –0.01)	–0.09 (–0.20 to –0.01)	–0.05 (–0.26 to 0.11)	–0.09 (–0.26 to 0.09)	–0.09 (–0.22 to 0.12)
LS mean (CI)	–0.09 (–0.17 to –0.01)	–0.11 (–0.19 to –0.03)	–0.06 (–0.16 to 0.04)	–0.05 (–0.15 to 0.05)	–0.03 (–0.14 to 0.08)
Y translation, mm					
Median (IQR)	0.04 (0.01 to 0.06)	0.05 (–0.01 to 0.12)	0.09 (–0.00 to 0.14)	0.10 (0.04 to 0.17)	0.09 (0.03 to 0.13)
LS mean (CI)	0.04 (0.02 to 0.07)	0.05 (0.00 to 0.09)	0.04 (–0.03 to 0.12)	0.04 (–0.05 to 0.14)	0.06 (–0.02 to 0.14)
Z translation, mm					
Median (IQR)	0.03 (–0.10 to 0.12)	–0.06 (–0.10 to 0.12)	0.01 (–0.23 to 0.10)	0.02 (–0.16 to 0.14)	0.04 (–0.15 to 0.11)
LS mean (CI)	0.08 (–0.15 to 0.31)	0.05 (–0.15 to 0.24)	0.04 (–0.17 to 0.26)	0.06 (–0.18 to 0.29)	0.04 (–0.22 to 0.31)
Total translation, mm					
Median (IQR)	0.19 (0.12 to 0.37)	0.28 (0.16 to 0.46)	0.31 (0.18 to 0.67)	0.32 (0.24 to 0.65)	0.33 (0.20 to 0.73)
LS mean (CI)	0.30 (0.17 to 0.44)	0.38 (0.22 to 0.54)	0.48 (0.31 to 0.65)	0.53 (0.33 to 0.73)	0.59 (0.40 to 0.78)
X rotation, °					
Median (IQR)	0.01 (–0.09 to 0.10)	–0.06 (–0.14 to 0.03)	–0.07 (–0.26 to 0.09)	–0.06 (–0.24 to 0.15)	–0.03 (–0.23 to 0.20)
LS mean (CI)	0.07 (–0.09 to 0.23)	0.03 (–0.15 to 0.21)	0.02 (–0.17 to 0.21)	0.04 (–0.16 to 0.23)	0.02 (–0.23 to 0.28)
Y rotation, °					
Median (IQR)	0.18 (–0.18 to 0.57)	–0.03 (–0.59 to 0.46)	–0.28 (–0.44 to 0.41)	–0.09 (–0.46 to 0.09)	–0.13 (–0.30 to 0.51)
LS mean (CI)	0.16 (–0.16 to 0.47)	0.04 (–0.24 to 0.32)	–0.04 (–0.27 to 0.20)	–0.05 (–0.29 to 0.19)	0.01 (–0.25 to 0.27)
Z rotation, °					
Median (IQR)	0.10 (–0.03 to 0.19)	0.11 (–0.00 to 0.22)	0.06 (–0.08 to 0.25)	0.11 (–0.02 to 0.23)	0.09 (–0.23 to 0.21)
LS mean (CI)	0.10 (0.02 to 0.18)	0.12 (0.04 to 0.19)	0.08 (–0.01 to 0.17)	0.07 (–0.03 to 0.17)	0.04 (–0.06 to 0.14)
Total rotation, °					
Median (IQR)	0.48 (0.33 to 0.77)	0.60 (0.43 to 0.91)	0.64 (0.40 to 0.86)	0.52 (0.28 to 0.97)	0.58 (0.39 to 0.96)
LS mean (CI)	0.61 (0.39 to 0.83)	0.70 (0.52 to 0.89)	0.70 (0.55 to 0.85)	0.65 (0.46 to 0.84)	0.76 (0.57 to 0.96)
MTPM, mm					
Median (IQR)	0.52 (0.40 to 0.86)	0.63 (0.40 to 0.95)	0.62 (0.44 to 1.07)	0.54 (0.37 to 1.26)	0.65 (0.43 to 1.21)
LS mean (CI)	0.62 (0.41 to 0.82)	0.74 (0.52 to 0.95)	0.82 (0.61 to 1.03)	0.83 (0.56 to 1.11)	0.95 (0.69 to 1.21)

CI: 95% confidence Interval, IQR: interquartile range, LS: least-squares, MTPM: Maximum Total Point Motion.

**Figure 5:**
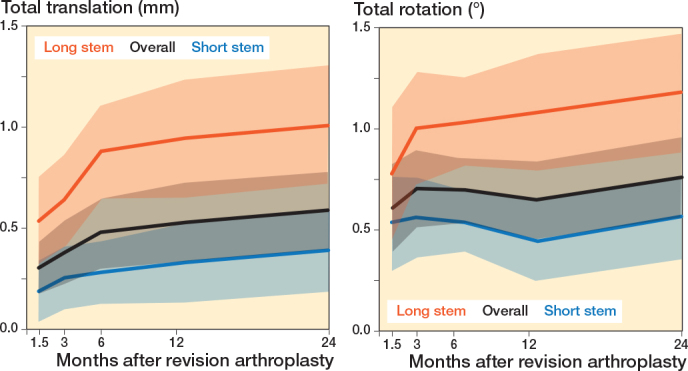
Least-squares means (LS means) with 95% confidence intervals derived from mixed models for repeated measures analyses, for total translation (left panel) and total rotation (right panel) over follow-up time. Red lines represents LS means for long stems, blue for short stems, and grey for all stems combined. Shaded areas indicate the corresponding 95% confidence intervals

Exploratory univariate regression analyses showed a significant association between stem length and TT and TR at 2-year follow-up (P = 0.001 and P = 0.002, respectively), constructs with long stems showing higher migration values compared with short stems. The LS means from the MMRM including stem length as covariate confirmed this pattern. At 2-year follow-up the TTs were 0.39 mm (CI 0.18–0.60) and 1.01 mm (CI 0.71–1.31) mm for short and long stems, respectively. The TRs were 0.56° (CI 0.35–0.76) for short stems and 1.18° (CI 0.89–1.47) for long stems (Figure 5).

### Clinical and functional outcomes

The PROMs and CROMs generally show improvement up to 6 months to 1 year postoperatively. VAS pain, OKS, KSS clinical and functional scores, and KOOS-PS all exceeded published MCID thresholds during this period (Table 4).

**Table 4:** Clinical and functional outcome scores presented as least-square (LS) means (95% confidence interval) at all follow-up time points

Item	Baseline	6 weeks	3 months	6 months	1 year	2 years
VAS						
Pain	7.0 (6.0–8.0)	4.3 (3.3–5.3)	3.2 (2.2–4.2)	2.8 (1.8–3.8)	3.8 (2.8–4.8)	4.0 (3.0–5.0)
Satisfaction	N/A	6.6 (5.6–7.7)	6.6 (5.5–7.8)	7.0 (5.9–8.1)	6.1 (5.0–7.2)	6.0 (4.8–7.1)
OKS	20 (16–24)	28 (24–32)	32 (28–36)	34 (30–38)	32 (28–36)	30 (25–34)
KSS						
Clinical	62 (56–68)	76 (70–83)	84 (78–90)	83 (77–90)	84 (78–90)	77 (71–84)
Functional	47 (38–56)	42 (33–51)	57 (48–66)	60 (51–70)	62 (53–72)	60 (51–70)
KOOS-PS	52 (45–59)	46 (40–53)	38 (31–45)	36 (29–43)	41 (34–48)	40 (33–47)

VAS: Visual Analogue Scale, OKS: Oxford Knee Score, KSS: Knee Society Score, KOOS-PS: Knee Injury and Osteoarthritis Outcomes Score – Physical function Short form, N/A: Not applicable.

Between 1 and 2 years, however, declines were observed in VAS pain, OKS, and KSS scores, with reductions exceeding their respective MCIDs, while KOOS-PS remained improved above MCID levels relative to baseline.

Although 2-year scores remained higher than preoperative values, this pattern suggests early functional gains followed by partial deterioration over time in this revision TKA cohort.

### Adverse events

**Figure 6:**
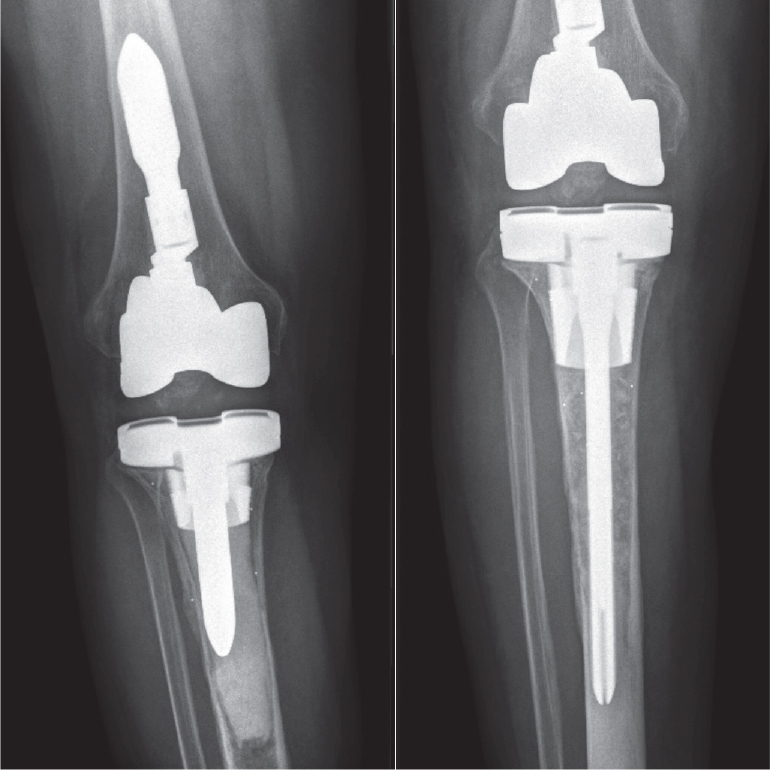
Anteroposterior radiograph of a patient with tibial loosening 14 days before re-revision surgery (left) and 6 weeks post re-revision surgery (right) after intraoperative confirmed tibial loosening. Before the initial revision surgery this patient had moderate epiphyseal and metaphyseal bone loss, mild diaphyseal bone loss, and the initial construct contained a tibial wedge, short cone, and a 120 mm tibial stem.

10 device-related AEs were recorded. 1 patient underwent a re-revision for aseptic loosening of the tibia component 12 months postoperatively (Figure 6). RSA analysis showed migration values for this patient of 1.14 mm TT and 0.95° TR at 1-year follow-up and ΔMTPM_6–12 months_ of 0.55 mm. This patient had moderate epiphyseal and metaphyseal bone loss, mild diaphyseal bone loss, and the removed construct contained a tibial wedge, short cone, and a 120 mm tibial stem. Another patient required an insert exchange due to instability, switching from a posterior-stabilized to a constrained insert. 6 patients reported persistent pain. Of these, 3 were referred to a pain treatment center, 1 received a local anesthetic (bupivacaine) injection at the painful anteromedial femoral region under ultrasound guidance (resulting in symptom relief), and 2 did not undergo further treatment. Additionally, 1 patient required physiotherapy for quadriceps atrophy, and 1 patient experienced balance issues due to a slight varus alignment for which an adjusted sole was prescribed.  

## Discussion

To our knowledge, this is the first RSA study to specifically evaluate the use of cones in rTKA. The primary aim of this study was to assess the stability of an rTKA construct utilizing metaphyseal cones. On a group level, RSA measurements demonstrated heterogenous early migration (until 3–6 months postoperatively). This was followed by limited additional migration at group level until 2 years postoperatively, suggesting satisfactory mechanical stability. However, several individual cases showed higher migration values of over 1 mm of translation (n = 3) or 1° of rotation (n = 6) at 2 years’ follow-up or showed a ΔMTPM_1–2year_ above the 0.2 mm threshold (n = 8) [25]. Importantly, these ΔMTPM thresholds were developed for primary TKA and should be interpreted cautiously in the context of revision TKA, where fixation conditions and construct complexity differ substantially. 1 patient underwent re-revision for tibial aseptic loosening (see Figure 6), and several other patients demonstrated migration values comparable to those of the re-revision patient. These findings highlight the variability in construct performance and the potential for clinical concern in these patients.

Direct comparison with existing literature is limited as only 1 RSA study involving rTKA compared hybrid-fixed with fully cemented implants [24]. Median migration values at 2-year follow-up of 0.27 mm TT and 1.06° TR were reported for the cemented group, and 0.31 mm TT and 0.52° TR for the hybrid-fixed group. Our 2-year migration values (TT 0.33 mm and TR 0.58°) are comparable in terms of translation and slightly lower in terms of rotation, considering that most constructs in our cohort were fully cemented. In the study by Mills et al. [24], using the same revision system in patients with less extensive bone loss (AORI type I and II), no cases of aseptic loosening were observed. The present group-level RSA estimates fall within a similar range, although differences in patient characteristics and bone loss severity limit direct comparability.

According to widely accepted standards for RSA in primary TKA the threshold for acceptable migration is an MTPM below 0.5 mm at 1-year follow-up [9], or a ΔMTPM_1–2-year_ of maximally 0.2 mm [25]. The observed value in this study was above the value considered “acceptable migration” (LS mean MTPM_1-year_ = 0.83 mm), but within the stability criterion of a ΔMTPM_1–2-year_ ≤ 0.2 mm (ΔMTPM_1–2-year_ = 0.10 mm). However, these benchmarks for primary TKA cannot be directly applied to revision TKA, where more migration would be expected based on less bone stock quantity and quality and very different implant constructs and fixation. This is underlined when comparing the present results with the recently published RSA benchmarks by Puijk et al. [26], who introduced a threshold of 0.3 mm migration at 6 months’ follow-up for primary cemented components. Using this threshold would leave 20 out of 25 of our revision components at risk of loosening. This stresses the need for validated RSA benchmarks for rTKA to guide clinical interpretation and identify constructs at risk of failure in this more complex setting.

Beyond RSA-based analyses, several studies have investigated the long-term survival of revision TKA using metaphyseal cones. Abdelaziz et al. reported a 10-year survival rate of 75% following rTKA [7]. They also observed a higher incidence of aseptic loosening in patients treated with pure hinged knee designs. Similarly, De Martino et al. found an overall 10-year rTKA survival rate of 81%, with a 96% survival rate specifically for aseptic loosening [8]. These findings are supported by other retrospective cohort studies reporting short- to mid-term survival rates of rTKA exceeding 90% [6,27,28], particularly in cases with significant metaphyseal bone loss. Such results underline the mechanical and biological advantages of metaphyseal fixation.

These observations raise an important clinical question: in which cases is the use of metaphyseal cones indicated? Although the current study was not designed to determine an indication threshold, over 75% of the cases presented with at least moderate metaphyseal bone loss according to the Belt classification. Importantly, the use of cones should be weighed against their relatively high cost and the desire to limit the amount of implanted material. Nevertheless, achieving stable initial fixation remains critical in reducing the risk of early failure and the need for complex re-revision procedures [2]. Striking the right balance between long-term implant stability and a conservative surgical approach is therefore essential in optimizing outcomes in revision TKA.

Prior research has shown that shorter (60 mm) cemented tibial stems used with cones provide comparable survival but superior functional outcomes compared with longer (100 mm) uncemented stems [29]. Similarly, another study evaluating the use of cones with and without stems found that while stems may slightly reduce micromotions, they are also associated with stress concentration at the stem tip and increased stress shielding, suggesting stems may not be essential [30]. In contrast, the present study utilized longer tibial stems (120 mm in 17 cases and 160 mm in 8 cases, mostly cemented). In exploratory univariate analyses, including MMRM-based estimates, constructs with longer stems demonstrated higher mean migration at 2 years. This may have contributed to the gradual decline in clinical and functional outcomes over time. This association should be interpreted cautiously, as longer stems are typically selected for cases with greater bone loss and poorer bone quality, indicating that the difference likely reflects underlying case complexity rather than a direct causal effect of stem length. Clinical outcomes peaked between 6 months and 1 year, followed by a gradual decline, which may also reflect the natural course of recovery and adaptation following rTKA.

### Limitations

First, to measure the stability of the construct comprising implant, stem, and cone, it was assumed that these components functioned as a single, stable unit. As migration of the tibial implant was measured relative to the bone markers, any theoretical micromotion occurring between the implant and the cone or stem could not be detected using this method. Second, due to the lack of a direct control group or established RSA migration thresholds for rTKA, interpretation of absolute migration values is limited. Third, conducting marker-based RSA in revision knee arthroplasty presents technical challenges. The limited availability and lower quality of remaining bone makes optimal placement of tantalum markers difficult, and radiographic visualization is often obstructed by the presence of long stems and cones. This can reduce measurement precision, and result in missing data at follow-up. Marker configuration models were used to mitigate these problems and to complete the analysis. In the future, CT-RSA may overcome these challenges by using CT-derived bone and implant models [31]. This eliminates the need for bone markers and implant models provided by the manufacturers. Fourth, the heterogeneity of the revision cohort should be acknowledged. The population included differences in stem and cone length, fixation method, use of augments or bone grafts, and insert types. Although this variation reflects the complexity of revision TKA in clinical practice, it may have introduced additional variability in the RSA migration data and limits the ability to isolate the effect of individual factors. Exploratory analyses suggested that stem length was associated with higher migration values, whereas cone length, use of wedges, bone grafting, and insert type did not show significant effects. These findings likely reflect both biomechanical differences and the greater bone loss typically present in cases reconstructed with longer stems.

### Conclusion

Our study demonstrates heterogeneous early migration with limited additional migration at group level from 1 to 2 years’ follow-up. However, the occurrence of higher migration in some cases, device-related adverse events, and the trend towards declining clinical outcomes over time underscore the need for extended follow-up and further comparative studies to optimize implant configurations and indications.

*In perspective*, although there was limited group-level migration from 1 to 2 years’ follow-up, the individual cases with higher migration indicate that fixation is not consistent across patients. Such findings support the use of RSA to guide postoperative surveillance in revision TKA. More long-term RSA data on revision TKAs is needed to establish meaningful benchmarks. Larger cohorts will be required to evaluate the influence of stem configuration, fixation technique, and augmentation strategy on migration patterns.

### Supplementary data

Supplementary Table 1 is available in Supplementary data on the article home page, doi: 10.2340/17453674.2026.45964

## Supplementary Material



## Notes

KM was responsible for Formal analysis, Investigation, Writing—Original Draft and Project administration. MB was responsible for Formal analysis, Investigation, Writing—Review and Editing, and Project administration. JHMG was responsible for Conceptualization and Writing—Review and Editing. GGvH was responsible for Conceptualization, Writing—Review and Editing and Funding acquisition. PJCH was responsible for the Conceptualization, Methodology, Writing—Review and Editing, Supervision and Funding acquisition.

The authors thank Saskia Susan for patient recruitment and study management and Jolanda Rubrech-van As for data management.

Handling co-editors: Li Felländer-Tsai and Robin Christensen

*Acta* thanks Maiken Stillling and other anonymous reviewers for help with peer review of this manuscript.
