# Supplementary material for: Early migration following revision total knee arthroplasty with tibial metaphyseal cones: a 2-year prospective RSA cohort study of 25 patients
Source: Acta Orthop. 2026 Jun 10;97:374–82. doi: 10.2340/17453674.2026.45964 (PMC13250721; doi:10.2340/17453674.2026.45964)
Supplement: Supplementary file 1 [file ActaO-97-45964-s1.pdf]

Supplementary Data

Table S1. Characteristics, migration values and clinical outcomes for patients with over 1 mm or 1° of TT and TR at 2-year follow-up

	013	049	064	085	109	113	047 [a]
Patient and implant characteristics							
Age	64	57	64	65	69	75	68
Body mass index	36.4	25.7	35.5	31.3	33.4	30.1	34.2
Revision stage (first/re-revision)	Re-revision	Re-revision	First revision	Re-revision	Re-revision	First revision	Re-revision
Bone loss [b] (epiphysis/metaphysis/ diaphysis)	3/2/1	2/1/0	2/1/0	2/2/0	1/1/0	2/2/0	2/2/1
Fixation method	Fully cemented	Fully cemented	Hybrid fixed	Fully cemented	Fully cemented	Fully cemented	Fully cemented
Tuberosity osteotomy	Yes	No	No	No	Yes	No	No
Tibial wedge	No	Yes	Yes	Yes	No	No	Yes
Insert type	Constrained	Constrained	PS	Constrained	Constrained	PS	Constrained
Cone length	Long	Short	Short	Long	Short	Short	Short
Stem length	Long	Long	Long	Long	Long	Short	Short
Migration (RSA)							
TT 1 year (mm)	1.36	1.50	2.17	0.53	0.75	0.27	1.14
TR 1 year (°)	1.01	1.57	1.88	1.49	1.20	0.47	0.95
MTPM 1 year (mm)	1.83	2.17	3.00	1.20	1.42	0.53	1.54
TT 2 years (mm)	1.33	1.82	1.95	0.62	0.88	0.97	N/A
TR 2 years (°)	1.17	2.22	1.72	1.52	1.06	1.24	N/A
MTPM 2 years (mm)	1.86	2.67	2.49	1.35	1.44	0.53	N/A
Clinical outcomes at 2-year follow-up							at 1-year follow-up
VAS pain	9	6	0	6	4	1	7
VAS satisfaction	10	Missing	10	5	5	0	3
OKS	13	30	16	31	29	39	28
KSS Clinical	55	69	99	70	64	95	69
KSS Functional	50	0	100	60	60	80	40
KOOS-PS	66.6	48.5	27.5	51.2	40.3	44.0	40.3

RSA: Radiostereometric Analysis; TT: Total Translation; TR: Total Rotation; MTPM: Maximal Total Point Motion; VAS: Visual Analog Scale; OKS: Oxford Knee Score; KSS: Knee Society Score; KOOS-PS: Knee Injury and Osteoarthritis Outcomes Score - Physical function Short form.

[a] This is the revised patient, who developed tibial aseptic loosening, the implant was removed after the 1-year follow-up; therefore, PROMs and CROMs are reported at 1 year instead of 2 years.
[b] Bone loss classification according to Belt et al. [15].
